# Traumatic hollow viscus and mesenteric injury: role of CT and potential diagnostic–therapeutic algorithm

**DOI:** 10.1007/s13304-020-00929-w

**Published:** 2020-12-19

**Authors:** Alessandro Michele Bonomi, Stefano Granieri, Shailvi Gupta, Michele Altomare, Stefano Piero Bernardo Cioffi, Fabrizio Sammartano, Stefania Cimbanassi, Osvaldo Chiara

**Affiliations:** 1 General Surgery Resident at Università Degli Studi di Milano, Milan, Italy; 2 General Surgery Unit, ASST Vimercate, Via Santi Cosma e Damiano 10, Vimercate, MB Italy; 3 Department of Surgery, University of Maryland, R Adams Cowley Shock Trauma Center, Baltimore, MD USA; 4 Chirurgia Generale e Trauma Team ASST Niguarda, Piazza Ospedale Maggiore 3, 20162 Milan, Italy

**Keywords:** Blunt trauma, Penetrating trauma, CT scan, Hollow viscus

## Abstract

Despite its rarity, traumatic hollow viscus and mesenteric injury (HVMI) have high mortality and complication rates. There is no consensus regarding its best management. Our aim is to evaluate contrast enhanced CT (ceCT) in the screening of HVMI and its capability to assess the need for surgery. All trauma patients admitted to an urban Level 1 trauma center between 2010 and 2018 were retrospectively evaluated. Patients with ceCT scan prior to laparotomy were included. Patients requiring surgical repair of HVMI and a ceCT scan consistent with HVMI were considered true positives. Six ceCT scan criteria for HVMI were used; at least one criterion was considered positive for HVMI. Sensitivity (Sn), specificity (Sp), predictive values (PV), likelihood ratios (LR) and accuracy (Ac) of ceCT of single ceCT criteria and of the association of ceCT criteria were calculated using intraoperative findings as gold standard. Therapeutic time (TT), death probability (DP), and observed mortality (OM) were described. 114 of 4369 patients were selected for ceCT accuracy analysis; 47 were considered true positives. Sn of ceCT for HVMI was 97.9%, Sp 63.6%, PPV 66.2%, NPV 97.6%, + LR 2.69, −LR 0.03, Ac 78%; no single criterion stood out. The association of four or more criteria improved ceCT Sp to 98.5%, PPV to 95.6%, + LR to 30.5. Median TT was 2 h (IQR: 1–3 h). OM was 7.8%—not significantly higher than overall OM. CeCT in trauma has become a reliable screening test for HVMI and a valid exam to select HVMI patients for surgical exploration.

## Introduction

Traumatic hollow viscus and mesenteric injury (HVMI) is uncommon in trauma patients, with an incidence of approximately 1.2% in blunt trauma and 17% in penetrating trauma [1–3]. This kind of injury recognizes different mechanisms: the most common is the crush between an object (i.e. seatbelt, steering wheel) and the spine posteriorly; rapid deceleration and burst injuries represent the other two main mechanisms. They can lead to local lacerations to the bowel wall and mesentery, mural and mesenteric infarction/hematomas, transection of the bowel, localized devascularization and full thickness contusions. Despite its rarity, HVMI seems to be related with higher mortality and complication rates compared to patients with similar injury severity score (ISS) without HVMI [2–4]. Currently there is no consensus regarding the best management of this injury; the choice between operative and non-operative management in hemodynamically stable patients remains difficult [4]. Common clinical parameters are not reliable and emergency room tests such as the extended focused abdominal sonography for trauma (E-FAST) are not appropriate for these injuries [4]. Moreover, the use of contrast-enhanced CT scans (ceCT) in stable patients is associated with a high rate of missing HVMI [5], due to a sensitivity of 80–96% and specificity of 48–84% [5]. A delay in treatment greater than 24 h has been shown to increase mortality, complications and length of stay [4, 5]. On the other hand, the low specificity of ceCT findings [6–9] leads to a high number of false positive cases with 30–40% of non-therapeutic laparotomies [4]. Many ceCT scan criteria have been described for HVMI diagnosis [10–13], but so far none has been associated with sufficient sensitivity and specificity when evaluated separately.

Given the complexity of diagnosing HVMIs, the primary aim of this study was to evaluate the use of ceCT scans in the screening of HVMI injuries and the capability of ceCT to assess the need of subsequent surgical interventions both in blunt and penetrating trauma. An algorithm is presented to assist the surgeon in the clinical decision-making.

## Methods

A retrospective analysis of trauma patients from the Trauma Registry of a Level I urban Trauma Center (ASST Niguarda, Milano) between October 2010 and August 2018 was conducted. The institution of trauma registry for all major trauma admitted to our trauma center was approved by the Niguarda Ethical Committee Milano Area 3 (record number 534-102018).

All blunt and penetrating trauma patients who received a pre-operative ceCT followed by laparotomy were included in the study. The multiphasic torso ceCT scan from the base of the skull to pubis was performed using a 64 detector multi-row scanner (Siemens Somatom Definition AS, Erlangen Germany), with injection of 1.7 ml/kg body weight of a 350 mg l/ml contrast agent at 3–4 ml/sec. A three-phase protocol including pre-contrast, arterial phase with trigger at 150 HU in the thoracic aorta, venous phase (70 s from trigger) was applied. The standard slice thickness was 1.2 mm at 1.0 pitch, with reconstruction at 1.2 mm and 2.5 mm. The first reconstruction was sent to the secondary workstation for multiplanar reconstructions and the second one to PACS. Attending radiologist performed and evaluated the exam.

Patients were divided in those with significant HVMI requiring surgical repair (full thickness perforation and/or bleeding and/or ischemic injury) and those without HVMI or with HVMI not requiring a surgical repair. Pre-operative ceCT was correlated with intraoperative findings. Patients with positive ceCT for HVMI and finding of HVMI requiring surgical correction at laparotomy were considered true positives; patients with ceCT negative for HVMI and no intraoperative finding of HVMI or with HVMI not requiring surgical correction were selected as true negative cases. To explore the effects of time-to-therapy on the outcome of patients with HVMI, patients who went straight to the OR without preoperative ceCT, because of hemodynamic instability, were included. These patients were excluded from ceCT accuracy analysis. Age, gender, type of trauma (blunt vs. penetrating), systolic blood pressure (SBP) and Glasgow Coma Scale (GCS) on admission in the emergency department (ED), AIS 98 score for each anatomic district (head, chest, abdomen, and extremities), ISS, TRISS calculated death probability and observed mortality were retrieved from the registry.

Six ceCT scan criteria for diagnosis of HVMI were selected: (a) free fluid without solid organ injury, (b) free intraperitoneal air, (c) gastrointestinal wall alteration (any focal anomaly of the bowel wall, including focal defect, thickening or thinning, abnormal or lack of enhancement with contrast), (d) mesenteric alteration (mesenteric hematomas and fat stranding), (e) intra-mesenteric fluid (accumulating between mesenteric layers and assuming a typical triangle aspect), (f) mesenteric blushing (active leak of intravenous contrast). Sensitivity, specificity, predictive values, likelihood ratios, and accuracy of ceCT and of the individual ceCT criteria were calculated using intraoperative findings as gold standard. The correlation between the number of ceCT criteria and HVMI requiring correction was analyzed to evaluate the capability of ceCT to assess the need of subsequent surgical intervention.

Data were collected in a computerized spreadsheet (Microsoft Excel 2016; Microsoft Corporation, Redmond; WA) and analyzed with a statistical software (IBM SPSS Statistics for Windows, version 25.0, IBM Corp., Armonk; NY). The sample distribution for all examined variables was evaluated with Shapiro–Wilk test. Differences in proportions were evaluated with Chi-Square or Fisher’s test when appropriate. Independent samples Mann–Whitney and Kruskal–Wallis tests were used to compare continuous variables. Results are reported as absolute values/percentages and medians/inter-quartile range (IQR). Based on the results of a previous large multi-centric study, time-to-therapy was recoded as a dichotomous variable, setting the cut-off at 8 h [2]. The incidence of Clavien–Dindo 3b postoperative abdominal complications was then compared between the two groups.

## Results

**Fig. 1:**
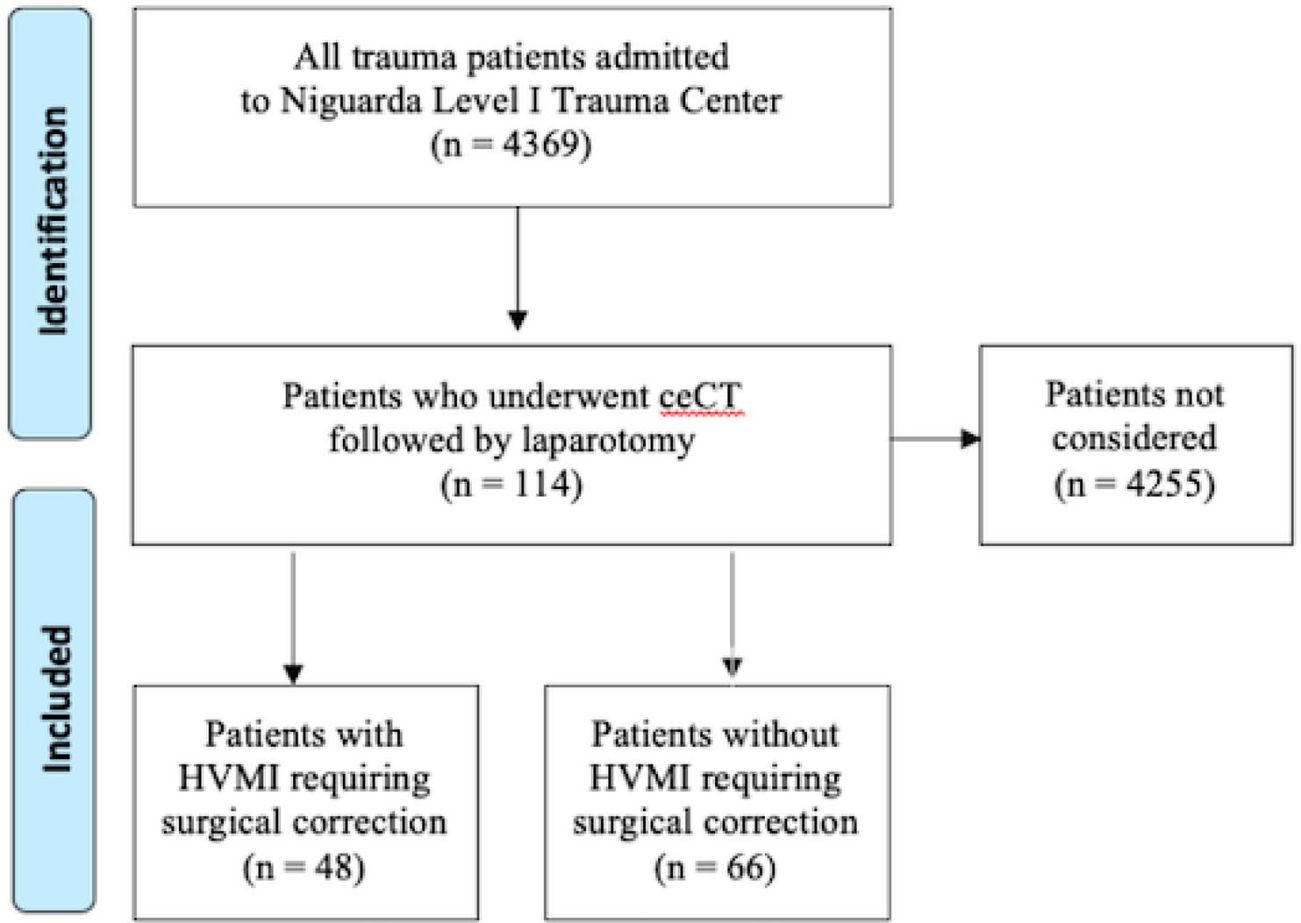
Flowchart of study design

Among 4369 trauma patients admitted between October 2010 and August 2018, 128 patients were identified according to the previously described modalities (Fig. 1).

Twenty-four patients (18.75%) sustained penetrating trauma. Fifty patients (39.1%) reported isolated small intestine/bowel/stomach injuries, whereas 14 patients had exclusively mesenteric injury. Ten patients (8.8%) sustained both hollow viscera and mesenteric injuries.

Of those, 14 patients were excluded from the ceCT accuracy analysis due to the need of emergent surgical intervention that prevented the possibility to perform ceCT scan. However, these patients were taken into account for time-to-therapy analysis (see below).

Of the remaining 114 patients, 48 patients had HVMI requiring surgical correction confirmed at laparotomy: 47 had a positive ceCT for HVMI and were used as true positives. One patient was a false-negative case because of a negative ceCT scan and a small bowel perforation at laparotomy.

The other 66 patients included 19 with HVMI not requiring surgical correction at laparotomy, 10 who underwent non-therapeutic laparotomies, and 37 who underwent surgical exploration for other indications and had no intraoperative evidence of HVMI. Among these, 42 had negative ceCT and were used as true negatives, while 24 had positive ceCT and were subsequently considered false positive. Descriptive analysis of study population is shown in Table 1. Among patients with HVMI, there were more males and more cases with severe thoracic injuries.

**Table 1:** Comparison between HVMI positive and negative groups

Variables	HVMI + (*n*.48)		HVMI – (*n*.66)		*p*
	Value	%	Value	%	
Gender (male)	44	91.7	48	72.7	**0.015**
Age (median/IQR)	41	31.75–52	39	23.5–57.5	0.899
Trauma (blunt)	36	75%	49	74.2	1.000
GCS (median/IQR)	15	14–15	15	14–15	0.664
SBP on admission (median/IQR)	125	103.75–140	110	91.25–133	0.112
ISS (median/IQR)	23.5	14–37.25	29	13.75–41	0.447
Head AIS ≥ 3	6	12.5	15	22.7	0.222
Chest AIS ≥ 3	21	43.8	43	65.2	**0.035**
Abdominal AIS ≥ 3	37	77.1	46	69.7	0.404
Extremities AIS ≥ 3	15	31.6	21	31.8	1.000

Most important results are indicated in bold

The values of sensitivity, specificity, predictive values, likelihood ratios and accuracy of ceCT (considered positive in presence of at least one criterion) and of single ceCT criteria for significant HVMI requiring surgical correction, are described in Table 2. Preoperative ceCT with at least one positive criterion showed a good sensitivity with a low level of false negative cases. The positive predictive value of ceCT progressively increased with the number of diagnostic criteria for HVMI. With four criteria or more, a HVMI requiring surgical repair was present in 95% of cases (Table 3).

**Table 2:** Values of contrast-enhanced CT (considered positive in presence of at least one criterion) and of the single ceCT criteria for HVMI requiring surgical correction (perforation, bleeding injury, ischemic injury)

	Sn	NPV	−LR (CI 95%)	Sp.	PPV	+ LR (CI 95%)	Accuracy
**ceCT**	**97.9%**	**97.6%**	0.03* (0.004–0.22)	**63.6%**	**66.2%**	**2.69 (1.95–3.71)**	**78%**
Free intraperitoneal air	35.4%	66.3%	0.69 (0.56–0.87)	92.4%	77.2%	4.25 (1.85–11,8)	66.6%
Free fluid without solid organ injury	75%	82.3%	0.29 (0.17–0.48)	84.8%	78.2%	4.95 (2.7–8.9)	81.5%
Intramesenteric fluid	45.8%	70.1%	0.58 (0.44–0.76)	92.4%	81.4%	6.05 (2.4–14.8)	71.9%
Blushing	43.7%	69.3%	0.6 (0.46–0.78)	92.4%	80.7%	5.7 (2.3–14.2)	72.8%
GI wall alteration	37.5%	65.9%	0.71 (0.56–0.9)	87.8%	69.2%	3 (1.4–6.5)	66.6%
Mesenteric alteration	72.9%	80.5%	0.33 (0.2–0.5)	81.8%	74.4%	3.63 (2.3–6.8)	75.4%

Most important results are indicated in bold

**Table 3:** Improvement of contrast enhanced CT ability to identify HVMI requiring surgical correction (perforation, ischemic injury, bleeding injury) with the increasing number of CT findings

	Sn	NPV	−LR (IC 95%)	Sp.	PPV	+ LR (IC 95%)	Accuracy
1 critierion	80%	97.6%	0.24 (0.042–1.43)	80.7%	28.5%	4.16 (2–8.4)	80.7%
2 criteria	72.2%	91.2%	0.32 (0.15–0.67)	86.6%	61.9%	5.4 (2.6–10.9)	83.3%
3 criteria	44.4%	76.1%	0.6 (0.39–0.91)	92.3%	61.9%	5.7 (2.1–15.5)	74.7%
** ≥ 4 criteria**	**45.8%**	**71.4%**	**0.55 (0.42–0.71)**	**98.5%**	**95.6%**	30.5* (22.3–37.5)	**76.3%**

Most important results are indicated in bold

Patients with significant HVMI (full thickness perforation and/or bleeding and/or ischemic injury) were more severely injured with a higher death probability compared to other trauma patients, while the observed mortality between the two groups was not significantly different (Table 4).

**Table 4:** Differences in injury patterns, death probability and mortality between patients with HVMI and all other trauma patients

Variables	Patients without HVMI (4307)		HVMI requiring surgery (62)		*p*
	Value	%	Value	%	
ISS (median / IQR)	9	4–21	27.5	15.5–41	** < .001**
AIS HEAD (AIS ≥ 3)	954	22.1	7	10.9	**0.032**
AIS CHEST (AIS ≥ 3)	1129	26.2	31	48.4	** < 0.001**
AIS ABDOMEN (AIS ≥ 3)	312	7.2	50	78.1	** < 0.001**
AIS EXTREMITIES (AIS ≥ 3)	825	19.1	24	37.5	** < 0.001**
TRISS death probability (median/IQR)	0.8	0.4–3.5	4.1	1.3–15.9	** < 0.001**
Observed mortality (median/IQR)	229	5.3	5	7.8	0.39

Most important results are indicated in bold

Effects of delay of treatment were investigated in 62 patients (48 true positive cases with preoperative ceCT plus 14 who underwent directly to OR for hemodynamic instability). Only one patient had a diagnostic delay greater than 24 h despite positive ceCT for HVMI, because the patient was admitted to an orthopedic ward. The median therapeutic time was 2 h (IQR 1–3). The majority of patients (55 out of 62) were taken to the OR within 8 h and 15 (27.3%) of those developed post-operative Clavien–Dindo 3b abdominal complications. Seven patients were surgically treated after 8 h or more and 5 (71.4%) developed postoperative Clavien–Dindo 3b abdominal complications. The comparison between the two groups showed a significantly higher proportions of complications among patients treated in delay (*p* < 0.01). Further details are reported in Table 5.

**Table 5:** Time-to-therapy and postoperative complications

	< 8 h (55 patients)		≥ 8 h (7 patients)		*p*
	Value	%	Value	%	
Clavien–Dindo 3b complications	15	27.3	5	71.4	< **0.01**
Acute renal failure	8	14.6	1	14.3	N.S
ARDS	10	18.2	1	14.3	N.S
Sepsis	9	16.3	2	28.5	**0.033**
Length of stay (median–IQR)	18.5	9–40	15	10.5–48	N.S

Most important results are indicated in bold

**Fig. 2:**
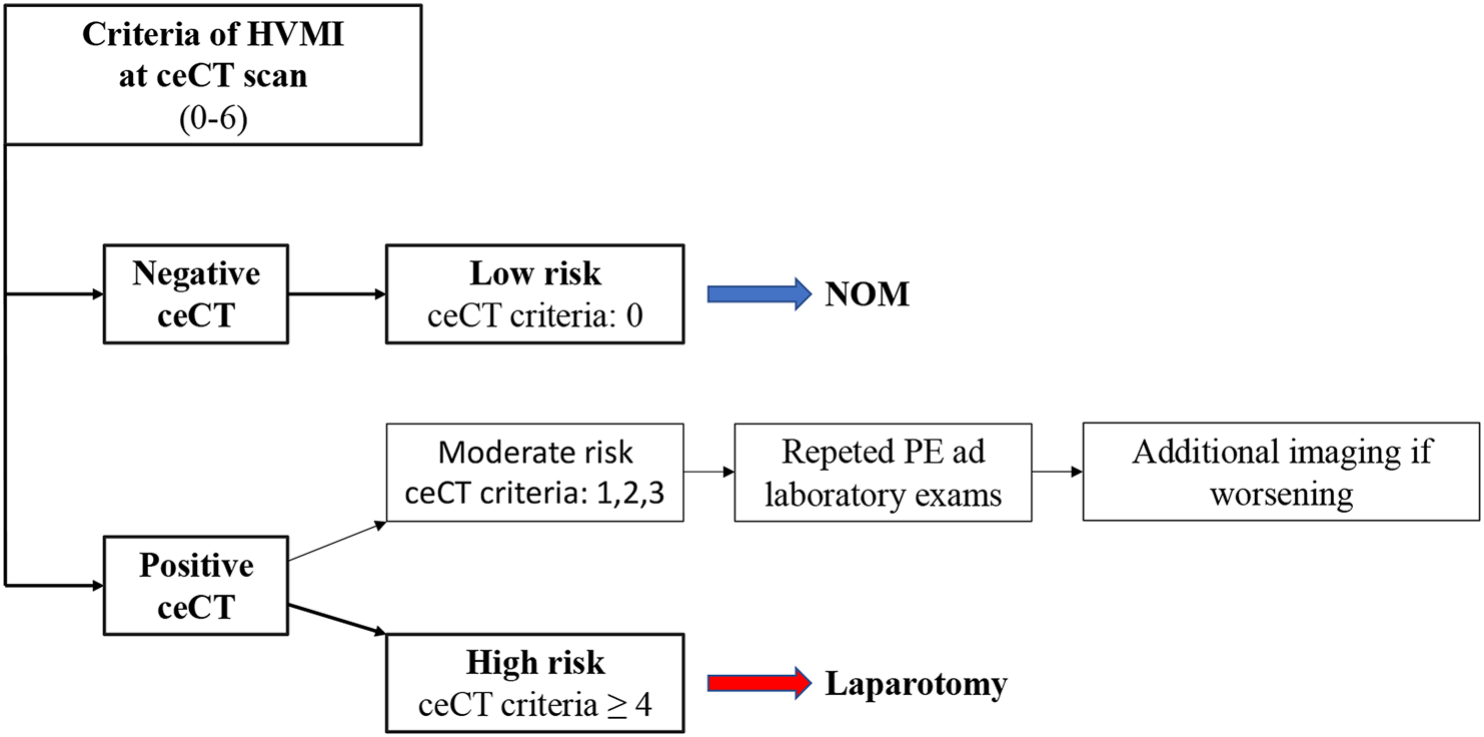
Potential algorithm for HVMI based on number of ceCT criteria

Finally, Fig. 2 shows an algorithm in trauma patients with suspected HVMI based on ceCT criteria. The number of ceCT criteria for HVMI can be used for the choice of treatment. An immediate laparotomy is suggested only in patients with four positive criteria or more. Patients with moderate or low risk are observed with serial physical examinations and laboratory studies. If any change is observed, a new ceCT is obtained and, if worsening, may be an indication to surgery. A completely negative ceCT allows observation only.

## Discussion

This study demonstrates that a ceCT without any sign of HVMI excludes the presence of this injury with a sensitivity of almost 98%. When the ceCT shows the presence of four or more diagnostic signs, a HVMI requiring surgical repair exists in 98% of cases.

Since 2000s, the nonoperative management of solid organ injury in hemodynamically stable patients became popular. The condition for this strategy was the exclusion with the ceCT of other injuries requiring laparotomy, such as a HVMI, the diaphragm rupture, a pancreatic injury with Wirsung duct transection. The ceCT, therefore, acquired a fundamental role in clinical decision-making of stable patients because of the higher morbidity and mortality of an abdominal missed injury treated in delay. Many studies evaluating the diagnostic accuracy of ceCT particularly for bowel injuries were published. A low sensitivity (87%) of ceCT for HVMI has been previously described in an Eastern Association of Surgery for Trauma (EAST) [5] large multicenter retrospective analysis based on more than 250.000 patients from 95 Level I Trauma Centers in the United States. Compared to the aforementioned trial, our study, despite a very similar proportion of isolated and combined hollow viscous/mesenteric injuries, reached more satisfactory results in terms of diagnostic accuracy.

Technology of computerized scans and radiologic expertise improved over the past 17 years and a growing number of studies [12, 14–18] showed that ceCT has become a reliable screening test for surgically relevant HVMI, able to exclude in the large majority of cases a HVMI both when discharging a patient from the emergency department and when dealing with the non-operative management of a solid organ injury [17, 19–21].

Although the false negative rate is less than 3% in many studies [20], 2.1% in our retrospective analysis, the limitation of ceCT still dictates a period of clinical observation until normal return of bowel function, particularly in patients with associated risk factors such as belted sign on the abdomen.

Although HVMI can be easily overlooked on a first superficial evaluation of ceCT, if any of the previously described ceCT criteria is identified during the diagnostic work-up, even if single and uncertain, the patient should be categorized as at high risk for significant HVMI and the Team Leader has to decide if a surgical exploration is indicated within the shortest time, ideally within 8 h and not past 24 h to avoid increased morbidity and mortality [14, 22, 23].

Our data confirm that ceCT had a low specificity for the immediate identification of significant HVMI (Table 3) and no single criterion was superior to the other, as shown in other studies [5].

However, the presence of multiple criteria greatly improved ceCT specificity, positive predictive value and likelihood ratio (Table 3). In particular, the presence of four or more criteria (accounting for 45.8% of all our HVMI cases requiring surgical correction) was associated with a specificity of 98.5%, PPV 95.6%, + LR 30.5. In this set of selected patients, the indication for emergency laparotomy is very reasonable and greatly surpasses the risk of non-therapeutic intervention without the need of further investigation.

In stable patients with fewer ceCT findings (1–3 criteria), the surgeon can start a non-operative management with period of careful observation, as suggested also by other authors [24, 25]. Changes in the clinical and laboratory picture (abdominal physical examination, increase in white blood cell count, lactate and amylase levels) should raise the suspicion of significant HVMI and suggest further investigations. Repeated imaging such as abdominal US [26] or a second ceCT [27], suggested as mandatory by some Authors after a standardized time from first evaluation [12, 28], may help in this set of patients to add valuable additional information, particularly useful in in non-assessable patients (spinal cord injury and/or impaired level of consciousness): if an increase to a significant number of positive criteria is reported, laparotomy should be again a reasonable indication.

In case of unclear clinical and imaging findings, laparoscopy may also help as a complimentary diagnostic tool. It must be underlined that laparoscopy is contraindicated in cases of hemodynamic instability and increased intracranial pressure. It is suggested in selected settings, such as patients with isolated free fluid without solid organ injuries and doubtful clinical examination [28], anterior abdominal stab wounds for peritoneal violation [29, 30], cases with urgent-emergent interventions other than laparotomy or in patients with anesthesiologic indication to intubation and sedation. In general, sensitivity and specificity of diagnostic laparoscopy are suboptimal and an experienced team is always recommended. Even if there are reports of the use of therapeutic laparoscopy [30], findings of HVMI as well as any doubt should warrant conversion to laparotomy.

More studies are needed to standardize how to clinically observe these patients, how to deal with changes in the clinical picture and if additional studies are reliable, taking also into account that late intestinal necrosis or perforation may develop many days after the initial trauma [31]. A number of studies proposed scores and algorithms [32–34] to continue to improve our ability to identify patients with significant HVMI and patients that are amenable to non-operative management. Our algorithm, represented in Fig. 2, is simple and based only on the number of criteria present at the ceCT scan. We deliberately avoided to include in our algorithm the dynamic of trauma (blunt vs penetrating), as well as other clinical features, to purpose a CT rather than a clinical-radiological score. Moreover, it is worth to underline that less than a fifth of the patients included in our study (18.75%) sustained penetrating trauma.

Limitations of our study reside in the retrospective nature of data collection and in the moderate-to-small number of patients with significant HVMI. Another limitation is that our control group included patients with other surgical indications and HVMI that did not require surgical correction. We also did not take into account the quantity and quality of free fluid without solid organ injury, where the number of Hounsfield Units, blood vs low density fluid such as bile, has been reported to be an accurate sign of bowel injury [35, 36]. Furthermore, the present study failed to obtain remarkable results for low risk patients. For them, our proposal of management relies more on the evidences available in the literature rather than on reported results.

In conclusion, combined with a tailored clinical observation, improving technologies and expertise have made ceCT in trauma both a reliable screening test to exclude significant HVMI and a valid exam to select the patients for surgical exploration when multiple criteria are represented.

Given the impact of these results on trauma patient management, further prospective studies are warranted to better define not only the diagnostic capacity of ceCT on HVMI but also the ability to correlate imaging results with proper therapeutic indication.
